# Case for diagnosis. Cicatricial alopecia on the vertex - Folliculitis decalvans and lichen planopilaris phenotypic spectrum^☆^

**DOI:** 10.1016/j.abd.2021.11.013

**Published:** 2023-03-07

**Authors:** Anna Carolina Miola, Paulo Muller Ramos, Hélio Amante Miot

**Affiliations:** a Department of Dermatology, Faculty of Medicine, Universidade Estadual Paulista, Botucatu, SP, Brazil; b Department of Dermatology, Faculty of Medicine, Universidade Estadual Paulista, Botucatu, SP, Brazil

Dear Editor,

A 34-year-old white female complained of vertex alopecia for six years, associated with pruritus and local burning sensation. She reported that, since the onset of the condition, she observed the development of pustules at the periphery of the lesion. She denied comorbidities or ongoing medication use. She reported having had cycles of trimethoprim-sulfamethoxazole for 60 to 90 days, with partial improvement. The examination disclosed cicatricial alopecia area on the vertex region of the scalp, with follicular pustules at the periphery, erythema and areas with polytrichia (Fig. 1). Dermoscopy revealed follicular pustules, polytrichia, perifollicular desquamation and erythema (Fig. 2). A biopsy of the peripheral region (area with disease activity) was performed, and histopathology showed a mixed perifollicular inflammatory infiltrate, rich in plasmocytes and histiocytes, affecting the region between the isthmus and the infundibulum, with follicular destruction (Figure 3, Figure 4).

**Figure 1:**
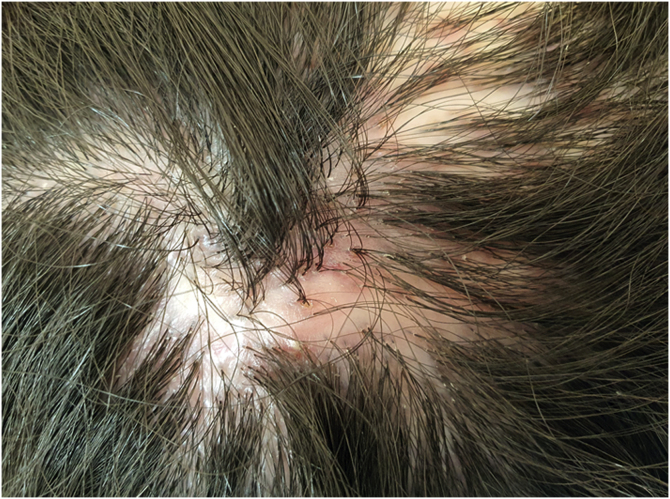
Cicatricial alopecia, with follicular pustules at the periphery, erythema and perifollicular desquamation, in addition to areas with polytrichia.

**Figure 2:**
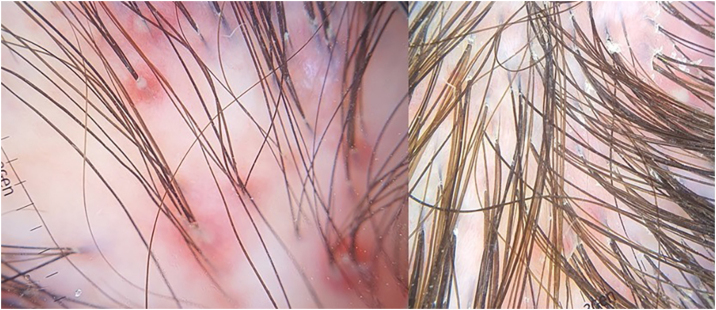
Follicular pustules, desquamation and perifollicular erythema on dermoscopy. Areas with absence of follicular ostia; however, with the presence of polytrichia.

**Figure 3:**
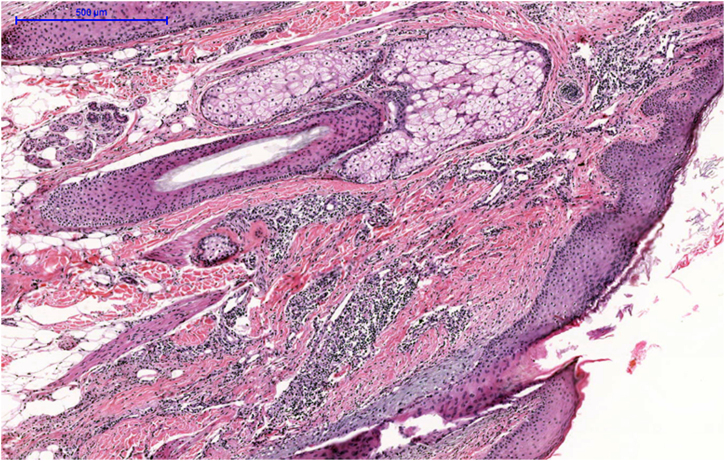
Mixed perifollicular inflammatory infiltrate in the region between the isthmus and the infundibulum, with follicular destruction (Hematoxylin & eosin, ×100).

**Figure 4:**
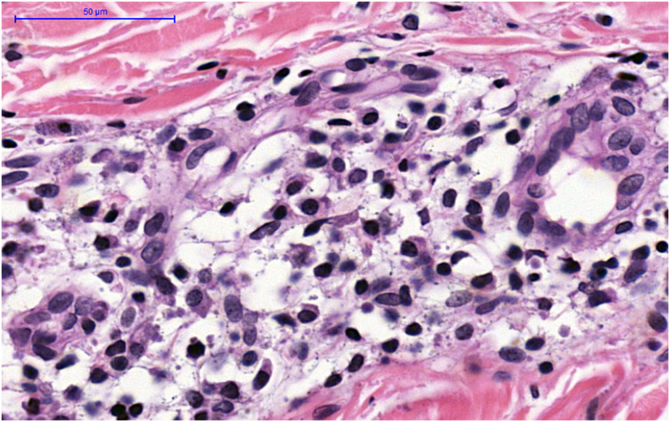
Presence of numerous plasma cells and histiocytes amidst the mixed perifollicular infiltrate (Hematoxylin & eosin, ×400).

## What's your diagnosis?

a) Folliculitis decalvans
b) Folliculitis decalvans and lichen planopilaris phenotypic spectrum
c) Centrifugal cicatricial alopecia
d) Lichen planopilaris

## Discussion

Folliculitis decalvans and lichen planopilaris phenotypic spectrum (FDLPPPS) is a recently described cicatricial alopecia, more prevalent on the vertex of the scalp in adults, which combines clinical and histopathological features of two other alopecias: lichen planopilaris (LPP) and folliculitis decalvans (FD), presenting with lymphocytic and neutrophilic inflammatory infiltrates, respectively.^1^

The pathogenesis of LPP and FD is still poorly understood; however, whereas in LPP there is activation of TCD8+ lymphocytes and loss of follicular immune privilege, in DF the presence of *Staphylococcus aureus* induces a biofilm that stimulates the innate immune response, perpetuating the neutrophil-mediated inflammatory process.^2^, ^3^

The etiology of FDLPPPS is still under discussion, given the coexistence (or alternation) of both inflammatory processes. It has been suggested that dysbiosis of the DF follicular microbiome may induce the exposure of follicular autoantigens, stimulating a Th1 response pattern.^1^, ^4^

As for the clinical picture, patients with FDLPPPS usually show a sequential biphasic process with the characteristics of FD preceding LPP, or even concomitant clinical characteristics: areas of cicatricial alopecia with follicular pustules, erythema and perifollicular desquamation, which may evolve with polytrichia, as in the present case.^5^, ^6^

Regarding histopathology, a mixed infiltrate is observed in the infundibulum region, with destruction and atrophy of the follicular epithelium and prevalence of plasma cells and histiocytes, in contrast to the predominance of neutrophils in the FD and lymphocytes in the LPP.^4^, ^7^, ^8^

There is yet no standardized treatment for FDLPPPS, given its recent description. However, treatments used in FD and LPP are usually associated, such as corticosteroids, sulfonamides, doxycycline, retinoid antimalarials, and immunosuppressants, according to the predominance of clinical and trichoscopic characteristics. In this case, oral hydroxychloroquine 400 mg/d and isotretinoin 30 mg/d, and clobetasol 0.05% gel were introduced, with stabilization of the condition after six months. The rationale for choosing anti-inflammatory drugs and oral retinoids was based on the predominance of clinical and trichoscopic signs of the LPP spectrum, in this case, mainly after previous treatment with antibiotics, aiming to reduce the neutrophil activity intensity.^1^, ^9^ However, it is noteworthy the lack of controlled studies on the efficacy of therapeutic strategies in cicatricial alopecia.

The cases described in adults and children with FDLPPPS do not seem to reach a large area of the scalp, despite the delay in its diagnosis.^1^, ^4^, ^5^, ^6^, ^7^, ^8^

Dermatologists should be aware of the diagnosis of FDLPPPS in the presence of less characteristic cases of cicatricial alopecia on the vertex of the scalp, with pustules, erythema and follicular desquamation, in which the inflammatory infiltrate is mixed, or with alternating patterns in subsequent biopsies, and containing plasma cells.

## Financial support

None declared.

## Authors' contributions

Miola AC: Design and planning of the study; collection, analysis and interpretation of data; critical review of the literature; critical review of the manuscript; writing and approval of the final version of the manuscript.

Ramos PM: Critical review of the literature; critical review of the manuscript, writing and approval of the final version of the manuscript.

Miot HA: Design and planning of the study; effective participation in research orientation; critical review of the literature; critical review of the manuscript, writing and approval of the final version of the manuscript.

## Conflicts of interest

None declared.
